# Hemolytic Uremic Syndrome: Toxins, Vessels, and Inflammation

**DOI:** 10.3389/fmed.2014.00042

**Published:** 2014-11-04

**Authors:** Victoria Cheung, Howard Trachtman

**Affiliations:** ^1^Division of Nephrology, Department of Pediatrics, NYU Langone Medical Center, New York, NY, USA

**Keywords:** thrombotic microangiopathy, hemolytic uremic syndrome, Shiga toxin, inflammation, alternative pathway of complement

## Abstract

Hemolytic uremic syndrome (HUS) is characterized by thrombotic microangiopathy of the glomerular microcirculation and other vascular beds. Its defining clinical phenotype is acute kidney injury (AKI), microangiopathic anemia, and thrombocytopenia. There are many etiologies of HUS including infection by Shiga toxin-producing bacterial strains, medications, viral infections, malignancy, and mutations of genes coding for proteins involved in the alternative pathway of complement. In the aggregate, although HUS is a rare disease, it is one of the most common causes of AKI in previously healthy children and accounts for a sizable number of pediatric and adult patients who progress to end stage kidney disease. There has been great progress over the past 20 years in understanding the pathophysiology of HUS and its related disorders. There has been intense focus on vascular injury in HUS as the major mechanism of disease and target for effective therapies for this acute illness. In all forms of HUS, there is evidence of both systemic and intra-glomerular inflammation and perturbations in the immune system. Renewed investigation into these aspects of HUS may prove helpful in developing new interventions that can attenuate glomerular and tubular injury and improve clinical outcomes in patients with HUS.

## Introduction

Hemolytic uremic syndrome (HUS) is characterized by generalized thrombotic microangiopathy (TMA) and the clinical triad of acute kidney injury (AKI), microangiopathic anemia, and thrombocytopenia (1). HUS is classified into three categories: (i) typical disease that occurs sporadically or in epidemic outbreaks and that is related to antecedent infection with Stx-producing strains of *E. coli* (STEC) or other microorganisms that elaborate Shiga toxin (Stx); (ii) sporadic atypical cases that occur secondary to infections, medication use, systemic disease, or malignancy; and (iii) familial atypical cases that are predominantly due to genetic abnormalities in complement regulatory proteins (1, 2) (Table 1).

**Table 1:** **Clinical taxonomy of HUS**.

	STEC–HUS	Atypical HUS non-familial	Atypical HUS familial
Cause	Shiga toxin producing bacteria	Infections Medications Malignancy	Genetic defects in regulation of alternative complement cascade
Incidence	1–3/10^5^ population	Unknown	1–3/10^6^ population
Need for RRT	40%	30%	50–60%
Mortality	3–5%	Depends on underlying disease	25%
Recurrence	Rare	Rare	25–50%
Progression to ESKD	<10%	Depends on underlying disease	50–70%

Hemolytic uremic syndrome is a perennial topic for review articles. Therefore, in an attempt to provide new insight into the disease, in this mini-review we will highlight the role of inflammation in both typical and atypical forms of HUS. Our intent is to indicate the potential of anti-inflammatory therapies to improve outcomes in patients with all forms of this disease.

## Epidemiology

All forms of HUS, taken collectively, qualify for the official rare disease designation, i.e., <200,000 cases per year in the US. The incidence of STEC–HUS has been steady and ranges from 6 per 100,000 in children under the age of 5 years to 1–2 per 100,000 in the overall population (1, 2) (Table 1). STEC–HUS disease affects girls more than boys, and occurs in all racial and ethnic groups except African Americans who are less likely to be affected (3). In epidemics, STEC–HUS may occur in a wider age spectrum. During the 2011 outbreak centered in Germany, 3,816 patients, predominantly adults, were infected by *E. coli* strain O104:H4, among whom there were 845 HUS cases, and 54 deaths (4).

The incidence of both non-familial aHUS and familial aHUS is lower than STEC–HUS. Together, they occur at a rate that is at most 10% of that for STEC–HUS and it is estimated that their total incidence is approximately 2–5 cases/million population per year (5). There is no increased susceptibility by gender or racial group for these two subcategories of HUS.

## Clinical Features of HUS

The presentation of HUS is usually abrupt and the diagnosis is made promptly. Patients develop pallor and noticeable oliguria within 1–2 days of the onset of the disease. STEC–HUS occurs approximately 6–14 days after ingestion of contaminated food or beverage and 2–6 days after the onset of enteritis. Diarrhea occurs in over 90% of cases of STEC enteritis. It is accompanied by severe crampy abdominal pain and stools that change from watery to hemorrhagic.

Non-familial forms of aHUS occur sporadically. Children with pneumococcal HUS tend to be young with a mean age of 1–2 years and have more severe disease compared to STEC–HUS. Up to 80% of affected patients require acute dialysis, compared to 40% in STEC–HUS; however, most children recover and have normal kidney function at long-term follow-up (5).

Patients with familial aHUS can present in childhood or adulthood (6, 7). They develop the disease throughout the year and at variable intervals after the triggering event. aHUS can be preceded by an episode of gastroenteritis, which can create uncertainty about the diagnosis.

Patients with HUS invariably have hypertension, hyperkalemia, and other electrolyte abnormalities such as hyponatremia and hypocalcemia as a consequence of the kidney injury. Nearly 40% of patients with STEC–HUS and up to 70% of cases of aHUS require renal replacement therapy during the acute episode (3, 6).

## Clinical Causes/Etiology

### Stx-producing strains of *E. coli*-hemolytic uremic syndrome

*E. coli* O157:H7 remains the most common strain that causes STEC–HUS (3). However, non-O157 *E. coli* serotypes are an increasingly prevalent cause of STEC–HUS. Between 2000 and 2006, these strains accounted for half of all STEC infections and the same percentage of isolates produced Stx2 as O157 strains (8).

### Non-familial aHUS

The most common infectious trigger is *S. pneumonia*, linked to neuraminidase production by the microorganism (5). The incidence has been steady because pneumococcal-related HUS is caused by bacterial strains, such as serotype 19A, that are not included in 7- or 23-valent pneumococcal vaccines (9). Other infectious causes include HIV, *Mycoplasma pneumoniae*, Histoplasmosis, and Coxsackie virus. It is important to note that STEC can also be associated with sporadic cases of HUS that resemble non-familial aHUS. Pharmacologic causes of aHUS include calcineurin inhibitors; antiplatelet drugs; chemotherapeutic agents, such as cisplatinum and gemcitabine; and biologics, such as the VEGF inhibitor, bevacizumab (10). SLE, the anti-phospholipid syndrome, and pregnancy can also lead to aHUS.

### Familial aHUS

Forty-four to sixty-six percent of patients with familial aHUS have one or more mutations in a complement regulatory protein (11). Combined mutations occur in <10% of cases and are more common in those with MCP or factor I mutations (12). The genetic defects in patients with aHUS generally demonstrate incomplete penetrance, indicating that additional factors are needed for the disease to be manifest. The presence of combined mutations or single mutations along with specific risk haplotypes is most significant for patients with MCP mutations because that subgroup had an increased risk of progression to ESKD and less favorable outcomes after kidney transplant (13). The gene that has been most recently linked to aHUS is diacylglycerol kinase ε (DGKE), an enzyme present in endothelial cells. The altered DGKE protein activated protein kinase C and caused a prothrombotic state (14). The impact of this protein on the alternative pathway of complement (APC) is unresolved because hypocomplementemia has been documented in a patient with a DGKE mutation (15).

## Pathophysiology

### Vascular injury

In HUS, the primary site of damage is the vascular endothelial cell with secondary injury occurring in the tubular epithelial cell and the podocyte. The endothelium becomes detached from the underlying basement membrane and the subendothelial space is filled with amorphous material and fibrin. Within the vascular lumen, there are platelet-fibrin thrombi that can completely occlude the vessel (16).

#### Stx-producing strains of *E. coli*-hemolytic uremic syndrome

When a person becomes infected with an STEC, bacteremia does not ensue. Instead, Stx crosses the gastrointestinal epithelium via a transcellular pathway, and enters the bloodstream (17). Stx binds to neutrophils, which convey it to the peripheral vasculature (18). Neutrophil-associated Stx is detectable in 60% of patients and the amount correlates with the extent of kidney injury (19).

In vascular beds, Stx binds to the glycosphingolipid, globotriaosylceramide (Gb_3_) on glomerular endothelial cells, mesangial cells, podocytes, and tubular epithelial cells (1, 2). The glomerular microcirculation is a key target of Stx because of high renal blood flow and enhanced expression of Gb_3_ but Stx also binds to the endothelium in other organs, especially the brain (20). After binding, Stx is internalized and transported to the Golgi apparatus and then in a retrograde pathway to the endoplasmic reticulum where it inhibits protein synthesis leading to cell death and apoptosis (21). The intracellular trafficking of Stx is blocked by manganese and administration of this cation protects against Stx-induced disease in experimental animals (22).

The vascular damage triggered by Stx promotes release of thrombin and increased fibrin concentrations. High levels of plasminogen activator inhibitor-1 (PAI-1) block fibrinolysis and accelerate thrombosis (23). Increased shear stress within occluded vessels perturbs processing of VWF multimers with uncoiling of the molecule. These alterations activate platelets and augment thrombus formation (23, 24). Angiopoietins are molecules that regulate angiogenesis by interacting with the Tie-2 receptor on the endothelial cell surface. Because of the pivotal role of endothelial injury in STEC–HUS, their role has been studied in this condition. Thus, children with STEC–HUS have reduced levels of angiopoietin-1 (anti-inflammatory) and increased levels of angiopoietin-2 (inflammatory) during the prodromal phase that worsen with progression of the microangiopathy. These alterations in endothelial function may potentiate systemic inflammation (25).

#### aHUS

In non-familial aHUS, injury to endothelial cells is directly caused by the offending agent. In familial aHUS, even slight endothelial damage during a mild viral respiratory illness can trigger TMA because of defective regulation of the APC (26). The alternative complement cascade is constitutively active because binding of C3 to factor B generates a catalytically active complex that leads to continued formation of C3bBb, the alternative pathway convertase. A number of circulating (Factors B, H, and I) and membrane-bound (MCP, thrombomodulin) molecules interact to prevent continuous activation of the pathway and endothelial injury (27). Both inactivating (Factor H and I) and activating mutations (Factor B and C3) enhance complement cascade activity and promote development of aHUS (6). Newly discovered mutations, including DGKE, have been associated with aHUS, but the contribution of APC activation in these cases is unresolved (14, 15).

### Inflammation

It has been evident from the time that HUS was recognized as a distinct clinical entity that inflammation plays a key role in the pathogenesis of the illness. However, it is important to recognize that the contribution of specific mediators is often limited by the small size of the patient cohorts, lack of serial sampling, and inability to test the impact of antagonists or inhibitors of the molecule of interest. Most of the literature on the inflammation in HUS is over a decade old, suggesting that this aspect of the disease has been largely neglected for the last 10 years. In the following paragraphs, the role of discrete components of the inflammatory response in the subcategories of HUS is summarized, primarily STEC-related disease. We highlight those studies that provide the strongest support for this pathogenetic pathway.

Stx-producing strains of *E. coli*-hemolytic uremic syndrome is associated with many inflammatory changes (Table 2) that may act as targets for new interventions. In a review of published literature, we were unable to identify studies that assessed these inflammatory processes in patients with aHUS. The exception is changes in complement, which have been explored in both types of HUS (7) (see below).

**Table 2:** **Inflammatory mediators in HUS**.

	STEC–HUS	Atypical HUS	Targeted therapies
Leukocytes	+++	+	None
Chemokines	IL-8	No data	None
	MCP-1	
	CXCR1	
	CXCR4/7-SDF-1	
Cytokines	IL-6	No data	?Anti-TNF-α agents
	TNF-α		?Anti-IL-6 agents
			p38 inhibitors
Complement	+	++++	Eculizumab

*?, Uncertain clinical value*.

#### Polymorphonuclear cells and monocytes

The addition of Stx2 to human bone marrow or cord blood cells in culture induces macrophage–granulocyte colonies (28). Polymorphonuclear (PMN) from Stx2-treated mice reveal increased expression of CD11b, greater adhesive properties, and enhanced cytotoxic capacity (29). Neutrophils contribute directly to renal inflammation and endothelial injury in HUS through the delivery of Stx to the glomerular microcirculation (18). PMN depletion reduces Stx2-induced cytotoxicity and renal damage (30).

In patients, who develop STEC–HUS, there is a 10-fold higher level of granulocyte stimulating factor, leukocytosis, and increases in absolute PMN and monocyte counts (31, 32). Based on biopsies done during the early phase of the disease, infiltration of the kidney by PMN is common in patients with STEC–HUS and atypical forms of the disease (24). Despite eventual PMN clearance from the kidneys after 2–3 weeks, a high peripheral blood PMN count at diagnosis is associated with a poor prognosis.

#### Chemokines

Strains of *E. coli*-hemolytic uremic syndrome is marked by the induction of various chemokines that recruit leukocytes to the kidneys. Mice exposed to Stx2 and lipopolysaccharide (LPS) showed higher levels of monocyte chemoattractant peptide-1 (MCP-1), macrophage inflammatory protein-1α (MIP-1α), and regulated on activation normal T cell expressed (RANTES), and increased macrophage recruitment to the kidney, compared to controls. Mice that were treated with neutralizing antibodies to these chemokines had decreased renal fibrin deposition (33).

Mice with Stx-induced HUS and knockout of chemokine (C-C motif) receptor 1 (CCR1) have improved survival, attenuated neutrophilia and monocytosis, reduced renal damage, and lessened PMN and monocyte renal infiltration compared to wild type animals. The rise in plasma tumor necrosis factor-α (TNF-α) and IL-6 levels was delayed in CCR1 knockout mice (34). The plasma concentration of these chemokines is acutely elevated in patients with STEC–HUS (35).

Stromal derived factor-1 is a potent chemoattractant cytokine that modulates stem cell mobilization, inflammatory cell infiltration, and angiogenesis via interaction with its receptor chemokine (C-X-C motif) receptor 4 (CXCR4). Treatment of human microvascular endothelial cells with Stx2 induced the expression of CXCR4, CXCR7, and stromal derived factor-1 (SDF-1). In a murine model of HUS, inhibition of the CXCR4/SDF-1 interaction decreased endothelial activation and kidney injury, and improved animal survival. Children infected with *E. coli* O157:H7 who progress to HUS have higher plasma concentrations of SDF-1, the endogenous ligand for CXCR4/CXCR7 chemokine receptor, compared to children whose enteritis resolves without complications (36).

#### Cytokines

Patients with STEC–HUS have higher plasma levels of TNF-α, IL-6, and lower levels of IL-10 compared to normal controls, a process driven in part by increased expression of Toll-like receptor on circulating PMN (37, 38). Isolated monocytes from children with STEC–HUS that are incubated with Stx1 produce IL-1β, IL-6, IL-8, and TNF-α (39). Preincubation of human endothelial cells with TNF-α or IL-1 results in an increase in Gb_3_ synthesis and increased Stx1 binding (40).

Circulating levels of TNF-α and IL-6 correlate with the severity of STEC–HUS and the occurrence of extra-renal complications. Similarly, monocyte production of TNF-α and IL-10 increases in parallel with the intensity of disease in children with STEC–HUS. Patients with moderate-to-severe disease had the greatest number of TNF-α producing monocytes (38). Patients who progressed to severe renal dysfunction had a 10-fold increase in IL-6 compared to those who maintained diuresis (41). Elevated concentrations of select cytokines, IL-6, and soluble TNF receptor 1, were associated with the occurrence of encephalopathy (42).

#### Complement

In a subset of patients with STEC–HUS, low levels of C3 have been documented in conjunction with increased levels of complement degradation products (C3b, C3c, and C3d) and deposition of complement components in kidney tissue (43, 44). A retrospective analysis of 17 patients with STEC–HUS found evidence of APC activation, based on high levels of factor Bb and soluble C5b-9 in the plasma, in all cases (3, 45). There is complement activation on platelet–leukocyte complexes and platelet- and monocyte-derived microparticles in the circulation of children with STEC–HUS (46). *In vitro* incubation of microvascular endothelial cells with Stx and murine models of STEC–HUS causes increased expression of P-selectin and activation of C3 via the complement cascade (47). Stx induces podocyte injury in mice via the activation of the APC and generation of C3a (48).

Patients with aHUS have reduced serum complement levels at diagnosis. In a study of 15 cases, decreased serum C3 level was noted in 73% of affected patients. A low C3 was associated with relative risk of developing HUS of 16.6 within families and of 27.8 in the overall population (12). Nearly 50% of cases of familial aHUS are due to activating mutations in proteins in the APC (e.g., C3, Factor B) or inactivating mutations in proteins that prevent overactivation of this cascade (16). It is worth noting that the same genetic abnormalities that cause aHUS have been linked to the development of C3 glomerulopathy and membranoproliferative glomerulonephritis, indicating that they can also lead to glomerular inflammation (49). The role of complement in aHUS has been the subject of intensive investigation and the reader is referred to an excellent up-to-date review by Noris et al. (7).

## Treatment

### Stx-producing strains of *E. coli*-hemolytic uremic syndrome

Provision of adequate amounts of sodium-containing intravenous fluids during the prodromal phase may prevent activation of the coagulation cascade within the glomerular microcirculation and prevent progression of STEC enteritis to HUS (50). There is no proven therapy for STEC–HUS that reduces the need for acute renal replacement therapy, shortens the course, or improves long-term outcomes. Therefore, treatment centers on supportive management of AKI, anemia, hypertension, and fluid-electrolyte imbalances. Renal replacement therapy is advised if there is anuria for at least 24 h or oliguria (<0.5 ml/kg/h) for at least 72 h (51) (Table 3).

**Table 3:** **Overall approach to the treatment of children with HUS**.

	STEC–HUS	Non-familial aHUS	Familial aHUS
Intensive supportive care	+	+	+
Treat triggering illness	−	+	−
	Antibiotics of no value		
Eculizumab	?	−	++
Plasmapheresis	−	?	+
	No supportive data		Temporarily until start of eculizumab treatment

*?, Uncertain clinical value*.

The agent that has received the most attention recently as a potential treatment for STEC–HUS is eculizumab, a monoclonal antibody to C5a. In the midst of the large German outbreak of STEC–HUS due to O104:H4 in 2011, a Letter to the Editor of the New England Journal of Medicine described three children, all 3 years of age, with severe STEC–HUS that required dialysis, who were treated with eculizumab (52). Their neurological status, platelet count, and LDH improved dramatically after the first dose. Dialysis was discontinued within 16 days and all three children were discharged without neurological findings. German nephrologists received emergency authorization to administer the antibody for “off-label” compassionate use during the outbreak and a total of 328 patients with STEC–HUS received eculizumab (53). Furthermore, open label treatment of nine patients in France during the outbreak promoted more rapid recovery of renal and neurological function (54). In contrast, in a report describing 90 children during the outbreak, of whom 13 received eculizumab (together with plasma exchange in 7 cases), the vast majority recovered and only 1 child died. The authors concluded that O104:H4 STEC–HUS is comparable to the O157 serotype and that there is no need for novel treatments such as eculizumab (55). A randomized clinical trial is needed to define the place of eculizumab in the management of STEC–HUS.

### Atypical HUS

Administration of the eculizumab to 37 patients with aHUS and renal damage (17 with plasma-resistant and 20 with plasma-responsive disease) resulted in a significant increase in platelet count and achievement of a TMA-event free status in 80% of cases (56). Following FDA approval in September 2011, eculizumab has become the standard of care for all patients with aHUS. Discontinuation of eculizumab has been associated with recurrence of disease (unpublished observations) and Alexion (the manufacturer) recommends maintaining antibody therapy indefinitely. A recent report describes 10 patients with aHUS in whom eculizumab was stopped; over a cumulative observation period of 95 months, 3 patients relapsed within 6 weeks of discontinuation but the drug was promptly restarted with complete recovery (57). Patients with genetic forms of aHUS and unsuspected disease activity may demonstrate clinical improvement after switching from plasma therapy to eculizumab treatment (58). Finally, eculizumab enables successful kidney transplantation in those who progress to ESKD (59).

### Potential role of inflammation as a therapeutic target

Refocusing on the role of inflammation in HUS may prompt a broader conception of the clinical problem and alternative approaches to treatment. This aspect of glomerular injury has not been a recent focus of research in HUS. However, we propose that therapeutic agents can be developed to modify the inflammatory mediators discussed in previous sections in order to attenuate glomerular injury and prevent acute and chronic decline in kidney function. This could include antagonists of chemokines such as IL-8 and SDF or inhibition of cytokines that are elevated during the course of HUS, such as TNF-α and IL-6. There are therapeutic agents in clinical use and in varying stages of drug testing that can interfere with the inflammatory response such as JAK/STAT pathway and p38 inhibitors (60, 61). Furthermore, agents such as exogenous angiopoietin-1 or the sphingosine-1-phosphate receptor agonist FTY720 (fingolimod) may prevent the inflammatory response to primary endothelial injury (25).

Targeting inflammatory mediators is emerging as a common approach to treating a number of diseases. For example, monoclonal antibodies to TNF-α and IL-6 and inhibitors of p38 MAPK used to treat patients with rheumatologic disorders are currently being evaluated for treatment of glomerulonephritis. As TNF-α has been proven to mediate glomerular and extra-renal injury in STEC–HUS, these antibodies may also be beneficial in treating STEC–HUS (40–42). A recent review of the role of infection and autoimmunity in glomerulonephritis points out the connection between HUS and various forms of glomerulonephritis and supports our proposal that anti-inflammatory therapy may be beneficial in HUS (62). It is important to note, however, that based on published pre-clinical and clinical data (see above), anti-inflammatory therapy may have a greater role in STEC–HUS compared to aHUS (Table 2).

## Prognosis

### Stx-producing strains of *E. coli*-hemolytic uremic syndrome

The prognosis of STEC–HUS is generally good and most children recover fully without subsequent relapses (63). The mortality rate in children is <5% but higher in adults (64). Neurological events are the most frequent cause of death (20). Short-term risk factors for decreased renal function and poor outcomes (including death) are the severity of the initial disease (e.g., oligo-anuria fever, leukocytosis, and colitis) and the need for prolonged dialysis, i.e., more than 7–14 days (65).

In patients who survive, renal abnormalities are the most common long-term complication. Children with persistent proteinuria, i.e., urine protein:creatinine ratio >1, or hypertension 1 year after resolution of the episode are at greater risk of progression to CKD (66). In a meta-analysis of the long-term outcome of children with STEC–HUS, up to 25% of children have evidence of CKD including hypertension, proteinuria, or reduced GFR (67). STEC–HUS accounts for 3–5% of patients with end stage kidney disease in most registries. Children with STEC–HUS who require kidney transplantation are not at risk for recurrent disease.

### aHUS

The clinical outcome in children with non-familial aHUS is determined by the underlying disease (Table 1). In most cases, there is recovery of renal function without any permanent loss of renal function. The overall prognosis for patients with familial aHUS is worse than STEC–HUS (Table 1). In patients with familial aHUS, the mortality rate can reach 25% during the first episode and children have a nearly ninefold higher mortality rate compared to adults (11). Similar to STEC–HUS, in those who do not succumb to acute episodes of the disease, impaired renal function is the most common long-term complication. Thus, up to 70–80% of survivors progress to ESKD and require permanent dialysis. A higher percentage of adults compared to children with aHUS progress to ESKD after their first episode of disease (11).

The genotype of familial aHUS influences the clinical outcomes. Patients with mutations in Factor H and Factor I have a higher rate of progression to ESKD and a greater likelihood of recurrent disease in a renal allograft. In contrast, mutations in MCP are associated with a more favorable course and minimal risk of recurrent disease post-kidney transplantation (11, 26).

## Conclusion

Hemolytic uremic syndrome is a rare but important disease in pediatric and adult patients. Although the clinical syndromes – STEC–HUS and aHUS – overlap, there are significant differences in the pathogenesis of these illnesses. The basis of treatment is reversal of the abnormality triggering the episode of TMA coupled with intensive supportive care. In some cases, improved understanding of disease pathophysiology has resulted in marked improvements in care, e.g., eculizumab in familial aHUS. However, the mortality and morbidity resulting from the TMA syndromes continues to be substantial. It is anticipated that future research in HUS will result in more sensitive diagnostic methods to detect disease earlier in the course of the illness. We have attempted to expand the mechanism of disease in HUS beyond direct vascular injury and complement-mediated damage to include renal and systemic inflammation. It is hoped that widening the scope in this way will facilitate the identification of new disease pathways, prompt the design of therapeutic agents that impact critical steps in the pathogenesis of each subtype of HUS, and improve outcomes for patients with this rare but life threatening illness.

## Conflict of Interest Statement

The authors declare that the research was conducted in the absence of any commercial or financial relationships that could be construed as a potential conflict of interest.
